# Quantifying the technical-tactical diversity of elite tennis players during match-play

**DOI:** 10.3389/fspor.2025.1634573

**Published:** 2025-09-23

**Authors:** Zichen Zhao, Yixiong Cui, Miguel-Ángel Gómez, Shouxin Zong, Bing Qi

**Affiliations:** 1 Sports Coaching College, Beijing Sport University, Beijing, China; 2 School of Sports Engineering (China Sports Big Data Center), Beijing Sport University, Beijing, China; 3 Facultad de Actividad Física y del Deporte (INEF), Universidad Politécnica de Madrid, Madrid, Spain

**Keywords:** technical-tactical diversity, uncertainty, entropy, racket sports, performance analysis

## Abstract

**Aims:**

The study aimed to model the diversity in technical-tactical performance among elite tennis players during matches in relation to match outcome and gender.

**Methods:**

Match performance data of 236 singles matches from the 2023 Australian Open and US Open were collected. Standard entropies were calculated for five technical-tactical performance indicators (shot type, forehand/backhand groundstroke direction, first/second serve distribution) to reflect each player's technical-tactical diversity. The linear mixed model (LMM) was used to examine the effects of match outcome and gender on each performance category.

**Results:**

The results showed that there was no statistically significant interaction effect between match outcome and gender on the diversity of five performance indicators (*p* > 0.05). However, both match outcome and gender independently had significant main effects on shot type, forehand/backhand groundstroke direction, and first-serve distribution (*p* < 0.05, conditional R-squared = 0.17–0.70). Losing players showed higher diversity in shot type [Effect Size (ES) = 0.33, small] compared to winning players. Male players generally showed greater diversity in shot type, and forehand groundstroke direction (ES = 0.63–0.97, moderate) than female players, but less diversity in backhand groundstroke direction and first-serve distribution (ES = 0.62–0.70, moderate).

**Conclusions:**

These findings suggest that (i) the technical-tactical diversity may help describe match styles of tennis players, instead of serving as a reliable indicator for assessing match outcome; (ii) the stability of technique usage is more crucial than the diversity of techniques used for elite players; and (iii) male players should focus on improving the attacking ability of the backhand to achieve higher rankings, while female players with exceptional serving abilities should prioritize the stability of first serve.

## 1 Introduction

Tennis competition at the elite level is becoming increasingly fast-paced and intense (1), which necessitates players to excel in tactical-technical, physical, and psychological domains (2). Correspondingly, the significance of performance analysis in tennis has grown steadily (3), enabling the identification of targeted training methods and the generation of insights for match preparation through a data-driven approach (4).

Extensive research has documented the statistical aspects of the technical and tactical skills of tennis matches, focusing on (i) Key performance indicators associated with victory. Winning players demonstrate higher rates of serve and return points won (5), baseline points won, break points converted, the ratio of winners to unforced errors, and points won in 0–4 shot rallies compared to losing players (6–8); (ii) Variations in technical and tactical skills across performance levels. Higher-performing players display greater ball velocity (9, 10), superior decision-making (11), and anticipatory skills (12, 13), more sophisticated tactical knowledge (14), and better visual search strategies (12). (iii) Gender differences in matches. Male players hit groundstrokes, serve, move at higher average speeds, and use more ball spin than their female counterparts at the Grand Slam level (15, 16). Additional studies have analyzed different playing styles among tennis players through statistical indicators (17), contributing to match preparation and training guidance.

Tennis is a complex sport where each point occurs in a specific context through combinations of techniques and shot directions. Single-dimensional indicators cannot fully capture the features of match play. Therefore, it is crucial to assess the level of technical diversity and shot direction players may demonstrate in competitive scenarios.

Diversity represents the fact of many different types of things or people being included in something (18, 19). In this study, technical-tactical diversity refers to the richness of different techniques (such as drop shot, slice) and tactical strategies (such as different groundstroke direction distribution) employed by athletes during match play. Variability stems from the human body's mastery of a large number of degrees of freedom (20). The redundancy of degrees of freedom within the system enables organisms to adopt multiple strategies to accomplish any given task, which naturally gives rise to variability (21). Variability is deemed as essential elements to understand its dynamics. This study does not focus on the variability between points or games, but rather the overall richness of the technical-tactical repertoire used throughout the match.

Entropy, a concept originating from thermodynamics and later adopted in information theory, is used to quantify the disorder and uncertainty of a system (22, 23). A higher entropy value indicates greater uncertainty. Performance variability and uncertainty can be quantified by using entropy, which provides a way to assess the overall state of the system. Entropy has been applied as an indicator of complexity in various sports (24, 25). For instance, Moras et al. (26) have used non-linear measures of entropy to analyze movement variability in resistance training among elite rugby players. Silva et al. elaborated on the use of various entropy measures to examine performance variability in team sports, revealing the interactions that shape players’ and teams’ performances (27). In soccer, Martinez et al. employed spatial and temporal entropy to analyze the randomness in football passing networks, identifying varied entropy levels across teams (28). In badminton, Galeano et al. used standard and spatial entropy to analyze the strike position distribution (29). However, the application of entropy methodologies remains underexplored when considering qualitative and categorical variables, where the distribution of each behavior can unveil the degree of disorder for a player or a team performance (24). Tennis, being a complex sport, cannot be fully understood through single-dimensional or static statistical indicators, thus calling for more aggregated analysis using standard entropy.

Given this background, the present study aimed to model the diversity in technical-tactical performance among elite tennis players during matches, comparing the differences in technical-tactical diversity between winning players and losing players within both male and female players, and examining the differences between male and female players. Based on existing literature, we hypothesized that winning players would show lower diversity in shot type and higher diversity in groundstroke direction and serve distribution. Additionally, it was expected that male players would show higher diversity in shot type and groundstroke direction but lower diversity in first-serve distribution compared to female players.

## 2 Method

### 2.1 Sample and data collection

This study analyzed 266 singles matches (532 player observations) from the 2023 Australian Open and US Open (136 matches for males and 130 matches for females) by retrieving data from a publicly accessed match statistics website (tennisabstract.com). In total, 50 male professional players (Ranking of Association of Tennis Professionals: 1–82) and 50 female professional players (Ranking of Women's Tennis Association: 1–78) were included, and the number of matches performed by individual players ranged from 1–14. The data consisted of information about the shot type, groundstroke direction, and serve distribution performance of professional players. The data reliability was tested by the authors via randomly recollecting the data of five matches, and the Kappa statistics of 0.75 showed substantial agreement (30).

Meanwhile, additional data related to groundstroke performance (points won by Winner and Forcing Shots) for players entering quarterfinals (having played at least 5 rounds) were collected by retrieving match-to-match data from the official website (www.ausopen.com), which yielded a total of 304 singles match observations from the 2023–2024 Australian Open (170 male observations and 134 female observations for female).

### 2.2 Technical-tactical performance

An initial inclusion of technical-tactical aspects relevant to player diversity was conducted by consulting two professional tennis coaches at the ATP tour-level and a performance director of national tennis teams. After that, a review of the extant literature was completed to help determine the indicators (31). Finally, five technical-tactical performance indicators were extracted from player's match actions and events (see Table 1). Smashes are not frequently used in matches, especially the backhand smash. Therefore, the smash-in-shot type is no longer categorized into forehand and backhand.

**Table 1:** Technical-tactical performance indicator.

Indicator	Sub-category
Shot type (Forehand/Backhand)	Topspin and flat Slice and chip Dropshot Lob Volley Smash^a^
FH/BH groundstroke direction	Crosscourt Down middle Down the line Inside-out Inside-in
First/second serve distribution	Wide T Body

FH, forehand; BH, backhand; ^a^Forehand and backhand smashes are considered equally; groundstroke only include topspin and flat shots.

**Figure 1:**
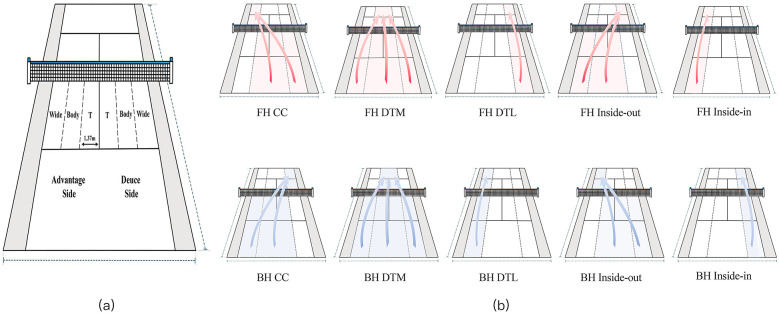
Illustration of serve and stroke directions. **(a)** The illustration of the service side (Advantage and Deuce) and service placement (Wide, Body and T); **(b)** groundstroke direction distribution; CC: crosscourt; DTM: down the middle; DTL: down the line.

**Figure 2:**
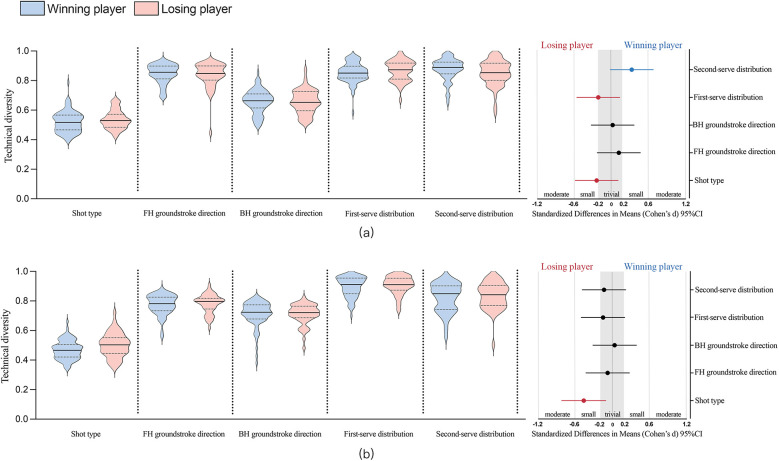
Diversity of technical-tactical performance in relation to match outcome and standardized (Cohen's d) differences in 5 technical-tactical performance variables. **(a)** represents male players and **(b)** represents female players; error bars indicate uncertainty in the true mean changes with a 95% confidence interval.

The graphical illustration of serve distribution and shot direction is showed in Figure 1. Crosscourt refers to shots from either the middle of the court or the far corner, hit to the opposite far corner. Down the middle is any shot hit to the middle third of the opposite court. Down the line are those starting in the left/right middle third of the court and bouncing in the opposite third. Forehand inside-out is played when a righted-handed player moves towards the left half of the court, with the initial objective of protecting his backhand to use the forehand drive, as shown by Figure 1B- ② (while backhand inside-out with shown in Figure 1C- ②). In the case of left-handed players, the forehand inside-out movement happens on the right-hand side.

Inside-out refers to shots starting in the middle of the corner, hit in the opposite direction of a crosscourt shot. Inside-in, almost exclusively forehands, are down-the-line shots where the player runs around a shot. Moreover, service placement is categorized as T (i.e., closer to the center service line), body (closer to the center of the service box), or wide (closer to the singles sideline of the service box) (32, 33). Specifically, serves landing on the boundary line between the body area and either the T area or the wide area was consistently classified as belonging to the body area. Similarly, any shots landing on the boundary lines of the middle area were classified as down-the-middle shots.

### 2.3 Standard entropy

Using data from five technical-tactical performance indicators, standard entropy was calculated for players at each match (24). This measure allowed controlling for variability in qualitative variables such as the diverse shot choices players make and different areas from which players serve in tennis and reflect the diversity in technical-tactical performance. This entropy can be calculated by dividing the Shannon entropy by the maximum value. In our case with 5 different technical-tactical performance indicators, the standard entropy, H, was calculated as follows:

$$\begin{array}{c} H=-\frac{\sum_{i=1}^{n}\;p_{i}*\ln\;(\;p_{i})}{\ln\;(n)}\#1 \end{array}$$

where *n* is the number of possible categories of technique in the variable and $p_{i}$ and represents the proportion of the usage frequency of each technique relative to the total usage frequency of all techniques. Consequently, the standard entropy lies in the interval from 0 to 1. A value close to zero would indicate that this player exhibited relatively few diversities in their game, while a value close to unity reveals a greater diversity.

### 2.4 Statistical analysis

The JAMOVI statistical package (version 2.4.11) was utilized to perform linear mixed model (LMM) to discern the difference of match outcome (win, loss) and gender (male, female) on technical-tactical diversity. LMM was apt for our design due to its proficiency in managing repeated measurements and encapsulating both fixed and random effects (ID for each player: repeated measure), enabling a nuanced analysis of intra- and inter-player variability.

Fixed effects were included for match outcome and gender, alongside their interactions to inspect potential factor-level dependencies. Random effects were attributed to players to account for individual differences.

To test the influence of fixed effect, we conducted an omnibus test. For assessing the impact of random effects, we employed the likelihood ratio test (LRT). We determined model fit with the REML criterion. The difference between fixed factors was measured using a *post hoc* test (Bonferroni). To elucidate interactions, estimated marginal means (EMMs) were computed for each fixed effects combination. To interpret the results of pair-wise comparison for practicality, effect sizes (ES) for all pairwise comparisons were defined, as follows: ≤0.2, trivial; >0.2, small; >0.6, moderate; >1.2, large; >2.0, very large; and >4.0, extremely large (32). The significance level was set at *p* ≤ 0.05 (34).

## 3 Result

Table 2 displays the technical-tactical diversity (mean ± SD) between male and female players.

**Table 2:** Mean ± SD for standard entropy of technical-tactical performance indicators.

Standard entropy	Male	Female
H shot type	0.52 ± 0.07	0.48 ± 0.07
H forehand groundstroke direction	0.84 ± 0.07	0.78 ± 0.07
H backhand groundstroke direction	0.66 ± 0.08	0.71 ± 0.08
H first-serve distribution	0.86 ± 0.07	0.90 ± 0.07
H second-serve distribution	0.87 ± 0.08	0.83 ± 0.10

Table 3 shows the linear mixed model's adequacy in fitting the standard entropy of technical-tactical performance indicators. Match outcome and gender have no statistically significant interaction effect on all 5 technical-tactical performances (*p* > 0.05). However, gender shows significant main effects on shot type, forehand/backhand groundstroke direction, and first-serve distribution (*p* < 0.05, r-square conditional = 0.17–0.70). The random effect likelihood ratio test (LRT) reveals that incorporating “player” (*p* < 0.01) as a random effect enhances the model fit. Omnibus test results indicate significant differences in shot type (*p* < 0.01), forehand groundstroke direction (*p* < 0.01), backhand groundstroke direction (*p* < 0.01), and first-serve distribution (*p* < 0.01) between genders, while 5 technical-tactical performance variables (*p* > 0.05) exhibit no differences in match outcome. The second-serve distribution shows no significant differences in both match outcome (*p* *=* 0.07) and gender (*p* *=* 0.30).

**Table 3:** The adequacy of linear mixed model.

Technical–tactical diversity	AIC	BIC	R2-marginal	R2-conditional	Estimate
Shot type	−749.55	−698.18	0.08	0.70	Yes
Forehand groundstroke direction	−679.00	−627.89	0.17	0.48	Yes
Backhand groundstroke direction	−602.74	−552.73	0.08	0.40	Yes
First-serve distribution	−701.25	−649.56	0.09	0.36	Yes
Second-serve distribution	−557.04	−507.49	0.04	0.29	Yes

AIC, akaike information criterion; BIC, bayesian information criterion.

Figuress 2,3 show the diversity of technique-tactical performance indicated among elite tennis players related to match outcome and gender, and standardized (Cohen's *d*) differences in five technical-tactical performance variables. Winning and losing players showed no statistically significant difference (*p* *>* *0.05*) among five technical-tactical performance variables (the ESs were unclearly trivial to possibly small). The diversity of shot type (*p* = 0.03, ES = 0.63, small to moderate) and forehand groundstroke direction (*p* < 0.01, ES = 0.97, moderate) for male players are higher than for female counterparts. The diversity of backhand groundstroke direction (*p* < 0.01, ES = −0.70, small to moderate) and first-serve distribution (*p* < 0.01, ES = −0.62, small to moderate) for female players are higher than for male counterparts. The diversity of second-serve distribution (*p* = 0.07, ES = 0.44, small) shows no statistically significant difference between males and females. Finally, a follow-up comparison shows that Top-10 male players have an even higher degree of diversity in forehand groundstroke direction (male: 0.87 ± 0.06, female: 0.77 ± 0.06, *p* < 0.01, ES = 1.43, large), while lower diversity in backhand groundstroke direction than Top-10 female players (male: 0.67 ± 0.08, female:0.72 ± 0.06, *p* = 0.02, ES = −0.77, moderate).

**Figure 3:**
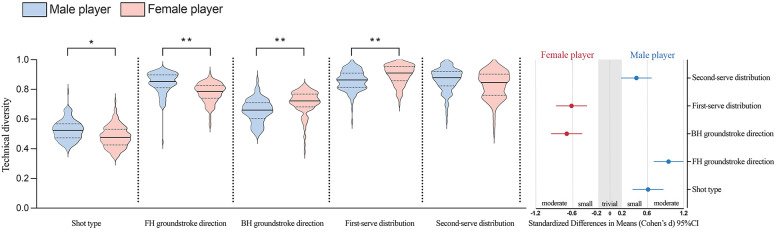
Diversity of technical-tactical performance in relation to gender and standardized (Cohen's d) differences in 5 technical-tactical performance variables. *p < 0.05; **p < 0.01; error bars indicate uncertainty in the true mean changes with a 95% confidence interval.

**Figure 4:**
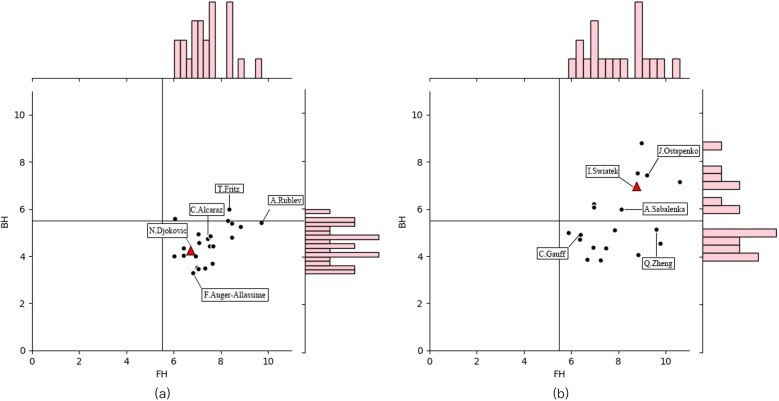
Forehand/backhand groundstroke winners and forcing shots for male and female players at the 2023–2024 Australian open. **(a)** represents male players and **(b)** represents female players.

Figure 4 shows forehand/backhand winners and forcing shots of groundstroke for male and female players at the 2023–2024 Australian Open. Forehand groundstroke won are higher than backhand for both male (*p* < 0.01, ES = 3.58, very large) and female players (*p* < 0.01, ES = 1.67, large). Female players exhibited higher backhand groundstroke won% (the proportion of forehand and backhand groundstroke won) than male players (male: 0.38 ± 0.09, female:0.41 ± 0.12, *p* *=* 0.01, ES = −0.33, small).

**Figure 5:**
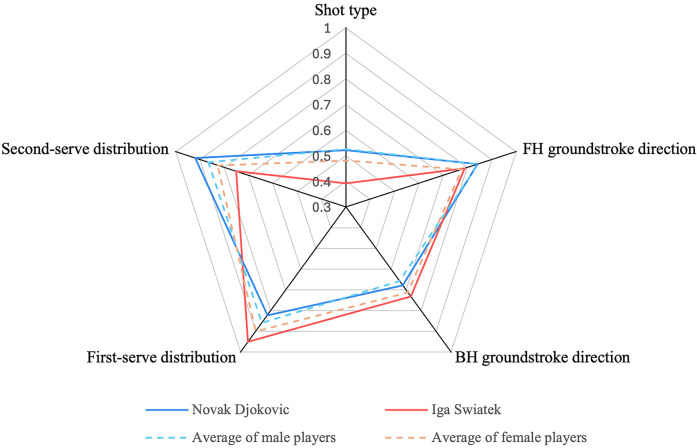
Average standard entropy of technical-tactical performance for novak djokovic, Iga swiatek, Top 100 male and female players in the 2023 Australian open and US open.

Figure 5 presents the mean of the standard entropy of technical-tactical performance indicators for the top 10 ranked male and female players and the rest. Both Novak Djokovic and Iga Swiatek distinctively showed no statistically significant difference from their peer players in all indicators (*p* > 0.05), but both players exhibited different diversity levels at their serve distribution.

## 4 Discussion

This study aimed to model the diversity in technical-tactical performance among elite tennis players during matches in relation to match outcome and gender. Losing players exhibited a higher level of diversity in shot type compared to winning players. Male players generally displayed a greater diversity in shot type and forehand groundstroke direction than female players, while showing less diversity in backhand groundstroke direction and first-serve distribution. These results support the assumption that performance in shot type, forehand groundstroke direction, and first-serve distribution is associated with match outcome and gender. Notably, female players showed higher diversity in backhand groundstroke direction than male players during match play, contrary to the initial hypothesis. These findings enhance our understanding of player performance during match-play and provide valuable insights for the training and development of elite tennis players.

### 4.1 Technical-tactical diversity in relation to match outcome

Results have demonstrated that the diversity of shot type shows no statistically significant difference between winning and losing players. On the one hand, due to the unique format of the Grand Slam tournaments, perhaps they had a clear arrangement of physical deposit and prioritized their serving and returning ability to minimize long rallies and avoid playing in precipitation, which would accelerate match fatigue (35). On the other hand, the percentage of 0–4 shot rally length (compared to 5–8 and 9+ shot rally length) in male players is approximately 70% in Wimbledon, while female players are 65% (7, 36, 37). Shot patterns are comparatively stable in 0–4 shots, and the way of gaining and losing points is relatively consistent. Therefore, the difference in the diversity of shot type between winning and losing players is minimal. Moreover, for elite players, the importance of the stability of technique usage outweighs the diversity of techniques used. Elite players often secure victories through their consistent and superior techniques or by continuously forcing the opponent's weakness during crucial moments (38), rather than depending on the diversity of techniques.

At the same time, no significant difference in the diversity of forehand/backhand groundstroke direction was found between winning and losing players. Points won of 0–4 shot rally length (i.e., short rally) were significantly correlated with success (7). Previous findings showed that in roughly 90% of matches, male players who excelled in short rally performance ultimately won the match at Roland Garros (7). The percentage of short rallies compared to medium-length and long rallies is significantly overwhelming. The findings also indicated a predominant occurrence of short points (in contrast to medium and long points) on grass courts for both genders, with around 66% for women and 72% for men of all points played concluding within fewer than 5 shots (7). Such a fact means that the pattern of the shot rally is relatively fixed, and players would aim for depth and the opponent's weakness instead of varying shot directions. Therefore, differences in the diversity of forehand/backhand groundstroke direction between winning players and losing players are hardly distinguished. Moreover, the study mainly used the summarized data of the whole match, which could not explain the player's tactical variation at each point. Consequently, a point-by-point analysis of shot direction should be taken by future research, along with factors such as speed, spin, and depth.

Results have demonstrated that there is no difference in the diversity of the first-serve distribution between the two match outcomes. The percentage of first-serve points won was significantly correlated with world ranking (39). Players in the study, who are in the top 100, possess excellent serving abilities and tend to serve within 15.27 cm of a service box line (40), which may explain the little difference in first-serve distribution between winning and losing players. Moreover, dividing the serving area into just three zones may not help to distinguish marginal differences. Serve velocity and serve points won are significantly decreased in losers while they are increased or kept constant in winners during the 5th set of the match (41). Extending this perspective, it is reasonable to assume that the diversity of serve distribution may also vary across sets. Therefore, future research could focus on set-by-set analysis and spatial-temporal data of ball bounces to analyze the serve distribution.

### 4.2 Technical-tactical diversity in relation to gender

Results have demonstrated that the diversity of shot type in males is higher than in females. Male players exhibit superior physical competence, attributed to systematic and scientific training and nutritional prescription (16) enabling them to run and swing the racket at higher speeds (42). It leads to a more intense and challenging competition, making victory more difficult to achieve. Consequently, it demands that male athletes employ a greater variety of technical combinations to execute strategic tactics during matches. Furthermore, due to the formidable defensive skills displayed by male players, scoring points solely through baseline attacks becomes a challenging task. It requires a continual effort to shrink the opponent's shot space, applying increased pressure. Therefore, it is more common to see male players attacking at the net to finish the points (43). However, Cui. et al. have demonstrated that female players had merely around 10% of total points won that were won in the net, which suggests that professional female players remained conservative in approaching the net and preferred baseline strategy in all Grand Slams to compete (44). In this context, coaches need to be aware of and understand the differences in diversity of shot type between men and women, so any expectations and goals set are realistic and sex-specific.

Male players generally had higher diversity in forehand groundstroke direction than female players, while lower in backhand groundstroke direction, particularly among the top 10 players according to the data collected in this study. Although the proportion of forehand points won is significantly higher than backhand for both sexes, male players tend to use more inside-out forehands in their offensive play. The forehand inside-out counts to 14% of the total shots of a tennis match (45). Specifically, these shots (forehand inside-out and inside-in) are mostly used as a stroke designed to induce a change of rhythm (45). In this scenario, male players predominantly rely on their backhand for rallies. Some female players excel more in backhand than in forehand. They tend to utilize backhands more frequently to change the direction of the ball. However, the current trend is towards an equal balance in forehand and backhand attacking capabilities, such as Novak Djokovic and Iga Swiatek. Therefore, male players should focus on improving the attacking ability of the backhand if they want to enter the top ranks.

The diversity of first-serve distribution in male players was lower than in female players. Gender differences in serve distribution indicated that male players showed a preference for serving towards the corners of the service box (40) and female players tended to serve more to the body than males (46). Serve is a more effective weapon for male players compared to female counterparts. Male players can gain an advantage or win points directly by fast and angled first-serves. Female players often utilize their serves as a means to initiate a point, rather than seeking to gain a direct advantage or win points outright (47). With female players typically generating lower serve speeds compared to male players, returners have more time to strategize and execute the serve-return, so points are less likely to be won directly from the serve (7). Female players enhance the threat of first serve by various locations of serve. Consequently, it may be prudent for coaches to focus on integrating the serve into a female player's holistic match strategy rather than aiming to win points directly from their serve. However, Aryna Sabalenka's diversity of first-serve distribution is lower than the average of female players (*p* = 0.05, ES = 0.80, moderate). She can gain an advantage or win points directly as men by fast and angled first serve. Therefore, some excellent female servers like Aryna Sabalenka should consider more about the stability of first serve in serve game.

Although the study provided a novel perspective to understand tennis player's performance, it is acknowledged that there are several limitations: (i) the study primarily relied on aggregated data from entire matches, which did not capture the tactical variations of players at specific points in the game; (ii) the samples are most high-ranked players from Grand Slam tournaments, and more analysis of lower-ranked and even junior players are needed; and (iii) the study only considered hard court and was unable to inspect the performance diversity on grass and clay courts; (iv) in Grand Slam tournaments, men's matches are contested in a best-of-five format, whereas women's matches follow a best-of-three format. This structure provides male players with more time to adapt to their opponents’ strategies. Consequently, as the match progresses, they are compelled to employ rarer techniques (e.g., drop shots or backhand down-the-line shots) to enhance tactical diversity and remain aggressive. The longer match format for men may result in higher entropy values compared to female players. To address this potential confounding effect, future studies should consider restricting the analysis of men's matches to the first three sets to control for bias introduced by the difference in match format.

## 5 Conclusion

In summary, the study revealed diversity in technical-tactical performance among elite tennis players during matches. All the five technical-tactical performance variables had no statistically significant difference in match outcome. Maybe diversity of technical-tactical performance was not an indicator to assess match outcome. It might be more suitable for describing player style. The higher diversity of shot type in males compared to females suggests that it is important for coaches to be aware that any expectations and goals set are realistic and sex specific. The higher diversity of forehand groundstroke direction and lower diversity of forehand groundstroke direction in males compared to females suggests that male players should focus on improving the attacking ability of their backhand if they want to enter the top ranks. The lower diversity of first-serve distribution in females compared to males suggests that female players with excellent ability of serve should consider more about stability of first serve in serve game. Future research will focus on a point-by-point analysis of technical-tactical diversity by using ball and player tracking data, integrating factors such as speed, spin, and depth.

### 5.1 Practical implications

- The technical-tactical diversity may help describe match styles of tennis players, instead of serving as a reliable indicator for assessing match outcomes.
- The stability of technique usage is more crucial than the diversity of techniques used for elite players.
- Male players should focus on improving the attacking ability of their backhand to achieve higher rankings, while female players with exceptional serving abilities should prioritize the stability of their first serve.
- We used standard entropy to quantify the technical-tactical diversity in tennis and provide a new perspective on understanding tennis player performance.

## Data Availability

Publicly available datasets were analyzed in this study. This data can be found here: http://tennisabstract.com.
