# Correction: Quantifying the technical-tactical diversity of elite tennis players during match-play

**DOI:** 10.3389/fspor.2025.1738474

**Published:** 2025-11-25

**Authors:** Zichen Zhao, Yixiong Cui, Miguel-Ángel Gómez, Shouxin Zong, Bing Qi

**Affiliations:** 1 Sports Coaching College, Beijing Sport University, Beijing, China; 2 School of Sports Engineering (China Sports Big Data Center), Beijing Sport University, Beijing, China; 3 Facultad de Actividad Física y del Deporte (INEF), Universidad Politécnica de Madrid, Madrid, Spain

**Keywords:** technical-tactical diversity, uncertainty, entropy, racket sports, performance analysis

Affiliation 1, “Sports Coaching College, Beijing Sport University, Beijing, China”, was erroneously given as “School of Competitive Sports, Beijing Sport University, Beijing, China”. Affiliation 2, “School of Sports Engineering (China Sports Big Data Center), Beijing Sport University, Beijing, China”, was erroneously given as “School of Sports Engineering, Beijing Sport University, Beijing, China”.

The original version of this article has been updated.

